# On Scars

**Published:** 1910-03-19

**Authors:** 


### Dermatology.

### ON SCARS.

The term "scars" includes any permanent mark left by past tissue changes. Scars are the legacies not only of violence, but also of the processes of disease. These comprise not only the various forms of ulceration, but also certain eruptions which, although unattended by actual ulceration, yet leave permanently visible traces on the skin. And when the opportunity occurs of examining the internal organs we find scars there too as the visible evidences of long past disease. There may be the fibrous knot left by an old focus of tubercle in the lung, or the depression resulting from a gumma in the liver. Scars are the monuments of pathological history. Clinically, much may be learned from their inspection, and frequently the information so gained is far more valuable than that afforded by the lips of the patient. If a crayfish loses a limb it promptly grows a new one; but if a similar misfortune befalls a mammal, all it can do is to form a protective fibrous scar over the wound, nor can it even reproduce the normal pattern of the skin if it be destroyed beyond a very trifling depth. The invariable rule is that all loss of tissue is replaced by fibrous tissue, and all varieties of epithelium are replaced by squamous epithelium.

The chief points to which attention must be paid in considering scars are their shape, consistency, level and colour. They may be linear, circular, oval or irregular in shape; they may be thick and rigid, or smooth and supple; they may be flush with the surrounding skin, or either raised above or depressed below its level. Further, they may be either pale or pigmented. Linear scars are always caused by external wounds, whether made by surgeons or others. Surgical cicatrices indeed are as a rule unmistakeable. They are linear, usually straight, sometimes, however, following a regular curve, and stitch marks may be distinguished at regular intervals on either side of the main scar. In cases where asepsis has been maintained, in course of time very slight traces indeed are left; and it is a further fortunate fact that, of all situations, it is on the face that the least conspicuous marks are left. To distinguish between scars made by surgical instruments and those made by weapons is, as a rule, not difficult. Wounds due to violence are frequently irregular and leave jagged scars. Bullets leave, as a rule, two scars—one at the point of entry and the other at the point of exit; usually they are merely circular depressions, but the wound at the point of exit varies very much according to the nature of the projectile. In many cases the position of the wound settles it to be a relic of surgery. Among the most frightful of all scars are those due to burns. Burns are often very extensive and, at the same time, very deep, and are therefore associated with great destruction of tissue. They are consequently slow to heal and there is liable to be an excessive formation of fibrous tissue. A scar from a bad burn is usually covered by thin atrophic epithelium very easily injured and covering a network of contracted fibrous cords. These, especially when occurring near a joint, may interfere seriously with the function of a part and cause marked deformity. The chin may be pulled down to the chest, the fingers to the palm, or the knee bent up to an acute angle by contracting scar-tissue following a bad burn. These unfortunate results may to a large extent be prevented by judicious surgical treatment, but nevertheless are far from uncommon. One curious point about burns is that, although they are slow to heal, the scars are seldom much pigmented.

All forms of ulceration leave scars, whether they be acute or chronic processes. In most cases the scars are characteristic. Everyone is familiar with the small, closely-set pits, massed together on the nose, more discrete on the cheeks, left by smallpox; while the pale, scattered and much shallower depressions after chicken-pox, though less conspicuous, are equally typical. Probably, however, in this country the most prolific sources of scars are tubercle and specific disease. Glandular abscesses, chronic sinuses resulting from bone and joint disease and lupus are the usual precursors of cicatrices. These scars are usually pale and atrophic, but where a sinus has existed for many years there is always considerable retraction, owing to the pulling of the fibrous cords formed along its track, and there is some pigmentation round the site of the orifice. The scars left by broken-down glands in the neck are generally irregular and unsightly. There is really no characteristic scar from lupus because so much depends on the treatment which has been adopted. There is no doubt that the best results are those obtained by the Finsen light, after which we find a supple, smooth skin, almost normal in appearance, and if the area of the disease has been small it is quite hard to discover its position. X-rays also give good results in suitable cases. They have the drawback, however, that there are liable to be a number of dilated blood-vessels in the scar. The occurrence of telangeiectases is always a risk attending the extensive employment of $x$-rays. The most unsatisfactory cases are those in which scraping has been repeatedly done and in which caustics have been used freely. In these there is much fibrosis, there are frequently unsightly tags of skin, and, as a rule, islands of the original disease to be seen still unsubdued.

In specific disease the most noteworthy point is the constant presence of pigmentation. Even after mild rashes which have never ulcerated, pigmented stains are frequently found, and the scars left after tertiary ulcers, although possibly pale in the centre, have a surrounding zone of smoky pigment. The consistency of these scars is commonly described as that of parchment. On the legs—a usual situation for these lesions—they often have to be diagnosed from varicose ulcers. The cicatrices from these, too, are usually pigmented; but there are certain points which, if attended to, should almost always enable a correct diagnosis to be made. Specific ulcers occur as a rule on the fleshy part of the limb, while varicose ulcers are found over the prominent parts of the bones. Single varicose ulcers may attain a very large size, specific ulcers, if large, are generally multiple. Varicose scars are almost uniformly pigmented, specific scars are much paler in the centre than at the periphery. Finally, where varicose ulcers have existed, enlarged and dilated veins and venules can always be found in the neighbourhood.

Of diseases which leave scarring without previous ulceration, the most important is lupus erythematosus. This is so marked a feature of this disease that the Germans call it "scar-erythema." Others are favus and the peculiar form of baldness, known as alopecia cicatrisata or the pseudo-pelade of Brocq. Of the diseases to which scar-tissue itself is liable, the commonest is that remarkable hypertrophy known as keloid. This may lead to the formation of tumours of considerable size. The negro races are particularly prone to suffer from this affection. Occasional cases of malignant disease arising in scars have been described.

